# Depressive Symptoms as a Novel Risk Factor for Recurrent Venous Thromboembolism: A Longitudinal Observational Study in Patients Referred for Thrombophilia Investigation

**DOI:** 10.1371/journal.pone.0125858

**Published:** 2015-05-04

**Authors:** Roland von Känel, Angelina Margani, Stefanie Stauber, Fiorenza A. Meyer, Franziska Demarmels Biasiutti, Franziska Vökt, Thomas Wissmann, Bernhard Lämmle, Paul S. Lukas

**Affiliations:** 1 Department of Neurology, Inselspital, Bern University Hospital, and University of Bern, Bern, Switzerland; 2 Department of Clinical Research, University of Bern, Bern, Switzerland; 3 Department of Psychosomatic Medicine, Clinic Barmelweid, Barmelweid, Switzerland; 4 Department of Cardiology, Swiss Cardiovascular Center, Inselspital, Bern University Hospital, and University of Bern, Bern Switzerland; 5 University Clinic of Hematology and Central Hematology Laboratory, Inselspital, Bern University Hospital, and University of Bern, Bern, Switzerland; 6 Center for Thrombosis and Hemostasis, University Medical Center, Mainz, Germany; 7 Privatklinik für Psychiatrie und Psychotherapie, Sanatorium Kilchberg, Kilchberg, Switzerland; IIBB-CSIC-IDIBAPS, SPAIN

## Abstract

**Background:**

Increasing evidence suggests that psychosocial factors, including depression predict incident venous thromboembolism (VTE) against a background of genetic and acquired risk factors. The role of psychosocial factors for the risk of recurrent VTE has not previously been examined. We hypothesized that depressive symptoms in patients with prior VTE are associated with an increased risk of recurrent VTE.

**Methods:**

In this longitudinal observational study, we investigated 271 consecutive patients, aged 18 years or older, referred for thrombophilia investigation with an objectively diagnosed episode of VTE. Patients completed the depression subscale of the Hospital Anxiety and Depression Scale (HADS-D). During the observation period, they were contacted by phone and information on recurrent VTE, anticoagulation therapy, and thromboprophylaxis in risk situations was collected.

**Results:**

Clinically relevant depressive symptoms (HADS-D score ≥8) were present in 10% of patients. During a median observation period of 13 months (range 5-48), 27 (10%) patients experienced recurrent VTE. After controlling for sociodemographic and clinical factors, a 3-point increase on the HADS-D score was associated with a 44% greater risk of recurrent VTE (OR 1.44, 95% CI 1.02, 2.06). Compared to patients with lower levels of depressive symptoms (HADS-D score: range 0-2), those with higher levels (HADS-D score: range 3-16) had a 4.1-times greater risk of recurrent VTE (OR 4.07, 95% CI 1.55, 10.66).

**Conclusions:**

The findings suggest that depressive symptoms might contribute to an increased risk of recurrent VTE independent of other prognostic factors. An increased risk might already be present at subclinical levels of depressive symptoms.

## Introduction

The etiology of incident venous thromboembolism (VTE), comprising deep vein thrombosis (DVT) of the leg and pulmonary embolism (PE), is multicausal, whereby genetic and acquired risk factors determine a person’s risk at a specific point in time [1]. Moreover, against a background risk from acquired prothrombotic conditions and inherited thrombophilia, several factors might facilitate a prothrombotic milieu resulting in the onset of VTE [2]. For instance, acute traumatic stress has been implicated as a potential triggering factor of PE [3]. Population-based studies revealed psychosocial factors like chronic perceived stress [4], low socioeconomic status (SES) [4–6], and depressive symptoms [7] as predictors of incident VTE, independent of demographic, life style, and clinical factors [4,5,7]. A plausible explanation for these links comes from abundant research showing that psychosocial stress, including depression, evokes hypercoagulability through autonomic and neuroendocrine mechanisms [8].

After a first episode of idiopathic VTE, the risk of another episode is highest in the first year (i.e., 10–15%) with a cumulative recurrence risk of about 25% at 5 years and 30% at 10 years [9,10]. Demographic and clinical risk factors of VTE recurrence differ considerably from those of incident VTE [11]. Unprovoked VTE, proximal DVT and/or PE increase the risk of VTE recurrence, while extended oral anticoagulation (OAC) therapy lowers the risk [12]. Other important risk factors for VTE recurrence include higher age, male sex, obesity, high levels of coagulation factors, and D-dimer levels ≥ 250 ng/ml after discontinuing oral OAC therapy [11–13]. Whether psychosocial factors are associated with an increased risk of VTE recurrence has not previously been investigated.

Compared with population norms, patients with VTE show impaired physical and mental quality of life (QoL) [14] and their mental QoL relates to depressive symptoms [15] and is lower than in patients with other forms of chronic venous diseases [16]. Up to 5 years after PE, some patients reported PE-related posttraumatic stress symptoms in the form of intrusive thoughts, flashbacks, and hypervigilance along with depressive symptomatology such as perseverative cognitions, social withdrawal, and tearfulness [17]. Posttraumatic stress after life-threatening events other than VTE is associated with depression and elevated clotting factor VIII activity [18,19], a predictor of VTE recurrence [20]. Likewise, in patients with VTE, negative emotions, including depression, have been associated with hypercoagulability [21,22]. Depression is also associated with poorer OAC management [23], age [24], sex [24], low SES [24], and obesity [25]. Depressive symptoms thus might be linked with recurrent VTE through demographic and clinical factors, psychological sequelae of VTE, as well as via direct effects on coagulation.

To the extent that prediction of recurrent VTE remains a clinical challenge [11], the aim of this study was to shed light on depressive symptoms as a potential and yet unevaluated risk factor. For this purpose, we assessed depressive symptoms in a cohort of patients with previous VTE scheduled for outpatient thrombophilia investigation. We hypothesized that higher levels of depressive symptoms at thrombophilia investigation would be associated with a greater risk of future recurrent VTE, whereby taking into account demographic and clinical factors previously shown to be associated with the risk of VTE recurrence and/or depressive symptoms.

## Materials and Methods

### Study participants and design

**Fig 1:**
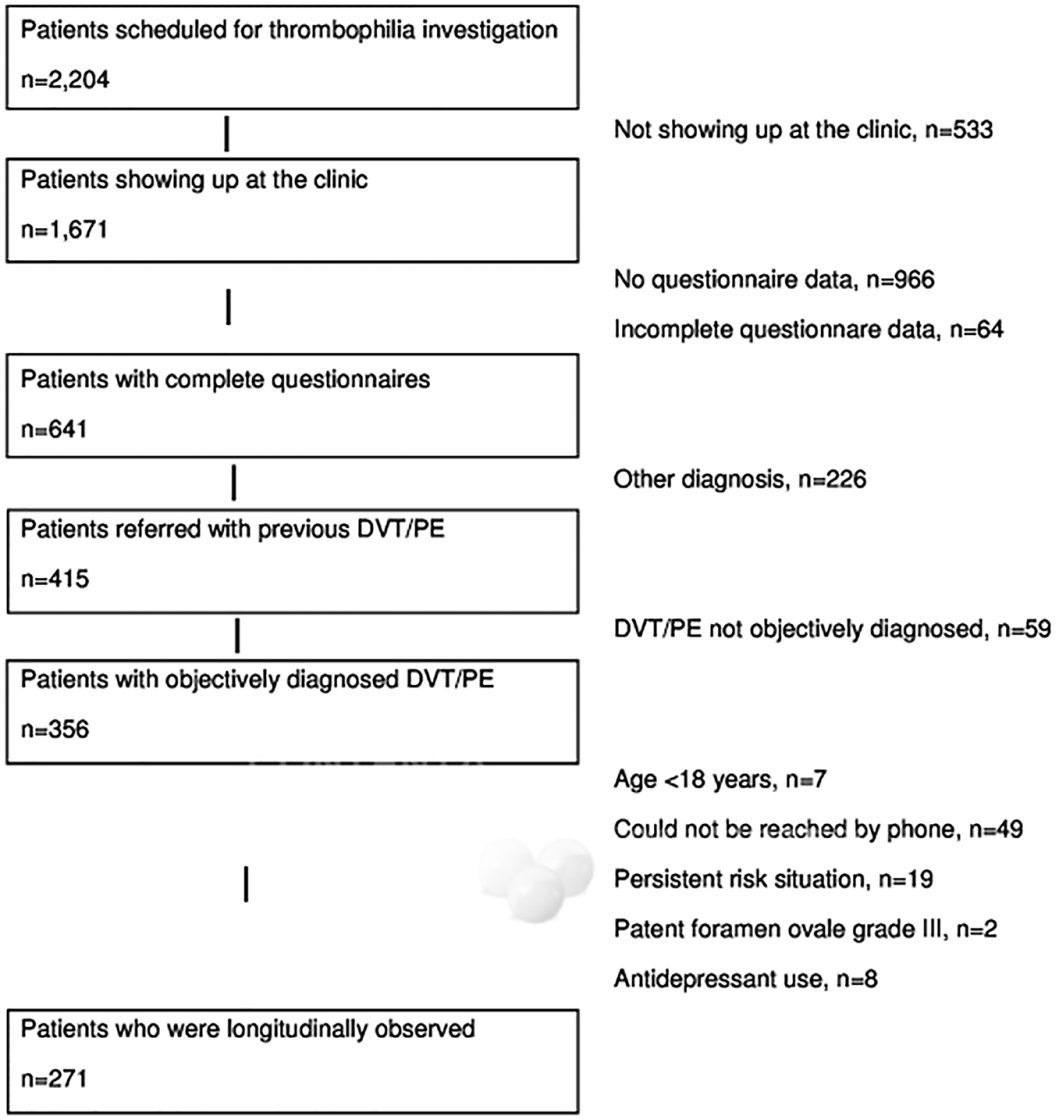
Flow chart of patient recruitment.

The participants of this longitudinal observational study were 271 consecutive patients who underwent thrombophilia investigation after an index episode of VTE at the outpatient ward of the University Clinic of Hematology and Central Hematology Laboratory, Bern University Hospital, Switzerland, between 03/2006 and 04/2011. For patients who had experienced more than one episode of VTE prior to referral for thrombophilia investigation, the most recent episode was defined as the index VTE event. The ethical committee of the State of Bern approved the study protocol. All participants provided written informed consent. The flowchart of the 271 patients investigated in the present study is shown in Fig 1, whereby the recruitment procedure of the original 2,204 patients scheduled for thrombophilia investigation has previously been detailed elsewhere [22].

Inclusion criteria for the current study were an objective diagnosis of the index VTE event, that is DVT of the leg and/or PE, age ≥18 years at thrombophilia investigation, and availability for follow-up assessment. Objective means to make a diagnosis of the index VTE event were compression ultrasonography, computed tomography (CT) or magnetic resonance imaging (MRI) venography, spiral CT, MRI pulmonary angiogram, catheter pulmonary angiography, or ventilation-perfusion scintigraphy. We included patients with a secondary cause for the index VTE event if they had a temporary thrombotic risk situation (provoked VTE): oral contraceptive use [26], pregnancy, surgery, physical trauma, plaster cast, immobilization, dehydration, and long-distance flight ≥6 hours. Exclusion criteria were persistent risk situations present at the time of the index VTE event; i.e., cancer, rheumatic diseases, multiple sclerosis [27], inflammatory bowel disease [12], organ transplantation, human immunodeficiency virus infection, and sex reassignment surgery with hormone replacement. We also excluded patients with a patent foramen ovale grade III, as recall of VTE recurrence could be obscured if VTE presents as paradoxical embolism. Those using antidepressants at thrombophilia investigation were also excluded, as antidepressant drugs are likely to influence depressive symptoms and hemostasis [28].

A questionnaire for the self-assessment of demographic factors and depressive symptoms was sent by regular mail to all patients 10 days prior to their clinical visit. Educational attainment was used to define SES (6 patients without graduation were grouped along with those indicating vocational education). Upon arrival at the hematology outpatient clinic, patients handed in the completed questionnaires. The hematologist obtained a history and asked about weight and height to compute the body mass index (BMI). Obesity was defined by a BMI ≥30 kg/m^2^. Additional information regarding the index VTE event, previous episodes of VTE, and current OAC was abstracted from medical records (note that in Switzerland, OAC at the time period of this study was almost exclusively established with phenprocoumon). Plasma D-dimer levels were measured with a quantitative sandwich enzyme immunoassay following the manufacturer’s instruction (VIDAS-D-Dimer, bioMérieux, Geneva, Switzerland).

### Assessment of depressive symptoms

We assessed symptoms of depression during the previous week with the validated German version of the depression subscale of the Hospital Anxiety and Depression scale (HADS-D) [29, 30]. The HADS-D has been widely used for the assessment of depressive symptom severity in somatic, psychiatric, and primary care patients, as well as in the general population [31]. To avoid any bias with symptoms from coexisting medical conditions, the HADS-D lacks items referring to somatic/affective symptoms of depression like sleep and appetite disturbances. The HADS-D is composed of 7 items each of which is rated on a 4-point Likert scale (0 = not at all, 3 = mostly; total sum score 0–21). Typical items are „I feel as if I am slowed down“; „I still enjoy the things I used to enjoy”(reverse coding). A score ≥ 8 defines the threshold for clinically relevant symptoms and identifies a major depressive disorder with a sensitivity of 82% and a specificity of 74% [32]. In the current sample, Cronbach’s alpha was 0.81, indicating good reliability of the HADS-D.

### Follow-up assessment

Following the original study protocol, trained and supervised PhD and medical master students aimed to contact patients by phone for a semi-structured interview twice; that is about 6 months and 12 months after the clinical visit for thrombophilia investigation, so to minimize the risk of recall bias. However, for logistical reasons and a shortage in staff resources, the observation period had to be extended to achieve two follow-up interviews, whereby 41.3% patients still could only be reached once. For all the patients, the observation period defined the time interval between the clinical visit for thrombophilia investigation and the last telephone call. An interview schedule was used to note patient indications with cross marks during the telephone interview.

Patients were asked about a new episode of VTE (DVT and/or PE) that had occurred since the clinical visit for thrombophilia investigation and since the first telephone interview (in those who had two phone contacts). In the case of a positive answer (yes), we requested hospital charts with confirmatory imaging test from treating physicians or institutions, whenever available, to establish a recurrent episode of VTE. Eligible imaging tests included compression ultrasonography, CT or MRI venography, spiral CT, MRI pulmonary angiogram, catheter pulmonary angiography, or ventilation-perfusion scintigraphy.

We further asked about anytime use of OAC drugs, compression stockings and/or low molecular weight heparin in risk situations since the clinical visit for thrombophilia investigation until the telephone interview (in those without VTE recurrence) or occurrence of recurrent VTE.

### Data analysis

Data were analyzed with SPSS version 21.0 for Windows (SPSS Inc., Chicago, IL) with significance level p<0.05 (2-tailed). We used the expectation maximization algorithm to replace a few missing HADS-D items (n = 18), and missing data for SES (n = 6), weight (n = 9), height (n = 6), D-dimer (n = 9), and time from the index VTE event to thrombophilia investigation/inclusion into the study/completion of the HADS-D subscale, subsequently termed “time since index VTE” (n = 2). Independent samples t-test with Levene’s test for equality of variances and chi-square test were used to compare groups with and without recurrent VTE on continuous and categorical variables, respectively. We calculated Pearson correlation coefficients to estimate the relationship between two variables.

We performed a binary logistic multivariable regression model with recurrent VTE (yes/no) as the outcome in relation to depressive symptoms as the predicting variable (note that we did not perform a survival time analysis because the time-to-event could not be verified by hospital charts in 22.2% of patients who reported a recurrent VTE event during the observation period). Covariate-adjustment was made for one block of variables with a p<0.20 on bivariate analysis associated with recurrent VTE. In a complementary model, we additionally entered covariates associated with depressive symptoms with a p<0.20 on bivariate analysis, but separately so to protect the model from being overfitted. We considered the following potentially important covariates based on their known associations with depressive symptoms and/or risk of recurrent VTE [11–13,21–25,33,34]: age, sex, SES, obesity, recurrent VTE vs. first-time VTE prior to thrombophilia investigation, proximal DVT and/or PE vs. distal DVT, unprovoked VTE vs. provoked VTE, D-dimer ≥250 ng/ml vs. <250 ng/ml, OAC at the time of the clinical visit for thrombophilia investigation (yes/no), as well as any OAC therapy (yes/no) and thromboprophylaxis in risk situations (yes/no) both since the clinical visit for thrombophilia investigation.

The primary analysis considered depressive mood as a continuous variable owing to literature showing that even minimal symptoms of depression, i.e., at subclinical levels, increase poor prognosis in patients after myocardial infarction [35]. We expressed the relative risk (odds ratio, OR) with 95% confidence interval (CI) for recurrent VTE in relation to a 3-point change on the HADS-D scale. This interval is clinically meaningful given the 3-point difference between the lower bounds of HADS-D scores to define mild (8 points), moderate (11 points), and severe (14 points) depressive mood [29, 30]. A secondary analysis considered depressive mood as a categorical variable to test for a potential threshold effect of depressive symptom severity on the risk of recurrent VTE. The Hosmer-Lemeshow test indicated good fit of all models. Cook’s distance assured the absence of undue outliers.

## Results

### Patient characteristics

Table 1 shows the demographic and clinical characteristics of the 271 patients categorized in two groups based on whether they had experienced a recurrent episode of VTE or not after thrombophilia investigation. During a median observation period of 13 months (range 5–46), recurrent VTE was reported by a total of 27 (10.0%) patients and objectively verified by chart records in 21 cases (no documented imaging tests available in 6 cases).

**Table 1:** Patient characteristics per recurrence of venous thromboembolism.

Variable	Recurrent VTE (n = 27)	No recurrent VTE (n = 244)	P-value
Index VTE event			
First-time VTE, n (%)	18 (66.7%)	184 (75.4)	0.322
Proximal DVT and/or PE, n (%)	17 (63.0)	160 (65.6)	0.787
Unprovoked VTE, n (%)	21 (77.8)	130 (53.3)	0.015
Time since index VTE (months)	8 (5–14)	8 (5–12)	0.615
Age (years)	47 (43–62)	47 (36–59)	0.084
Male sex, n (%)	18 (66.7)	128 (52.5)	0.160
Educational level, n (%)			
Graduate school (university)	16 (59.3)	95 (39.0)	0.119
Tertiary education	8 (29.6)	116 (47.5)	
Vocational education	3 (11.1)	33 (13.5)	
Body mass index (kg/m^2^)	26.9 (25.1–30.7)	26.4 (23.9–28.8)	0.204
Obesity, n (%)	7 (25.9)	50 (20.5)	0.511
D-dimer ≥250 ng/ml, n (%)	17 (63.0)	149 (61.1)	0.848
Current OAC therapy, n (%)	7 (25.9)	64 (26.2)	0.973
Depressive symptoms (score)	4 (3–6)	2 (1–5)	0.058
Clinically depressed, n (%)	2 (7.4)	25 (10.2)	1.000
Observation period			
Duration (months)	13 (12–27)	13 (12–24)	0.615
2 phone calls, n (%)	16 (59.3)	143 (58.6)	0.948
OAC therapy, n (%)	6 (22.2)	76 (31.1)	0.3838
Thromboprophylaxis, n (%)	22 (81.5%)	174 (71.3)	0.262

OAC, oral anticoagulation; DVT, deep venous thrombosis; PE, pulmonary embolism; VTE, venous thromboembolism. Data are given as number of observations (n) and percentage of total (%) or median with interquartile range. P-values refer to group comparison.

Unprovoked index VTE was significantly more frequent in patients with recurrent VTE compared to those without VTE recurrence. The prevalence of clinically relevant depressive symptoms, as defined by a cut-off on the HADS-D score ≥ 8, was 10.0% across the entire sample and similar in both patient groups. In more detail, depressive mood was mild in 7%, moderate in 2%, and severe in 1% of all patients (in the group with recurrent VTE, mild and moderate depressive mood were present in one patient each). Yet, there was marginal significance for a higher level (mean ±SD) of continuously measured depressive symptoms in patients with compared to those without recurrent VTE (4.41±2.27 vs. 3.24±3.05, p = 0.058). All of the other characteristics, assessed at the clinical visit for thrombophilia investigation and during the observation period, did not significantly differ between the two patient groups.

### Associations with depressive symptoms

Depressive symptoms did not significantly associate with characteristics of the index VTE event, namely first-time VTE vs. recurrent VTE (p = 0.70), proximal DVT and/or PE vs. distal DVT (p = 0.29), unprovoked VTE vs. provoked VTE (p = 0.98), and time since index VTE (p = 0.86).

In terms of demographic and clinical factors, depressive symptoms showed no significant relationship with SES (r = -0.12, p = 0.051), age (p = 0.32) and sex (p = 0.55). More depressive symptoms were significantly associated with obesity (r = 0.13, p = 0.040), but not with elevated D-dimer (p = 0.93), even when controlling for current OAC use (p = 0.67).

Regarding the observation period, depressive symptoms were significantly associated with greater use of OAC (r = 0.14, p = 0.026), but not with the duration of the observation period (r = 0.11, p = 0.074), the number of phone contacts (p = 0.50), and thromboprophylaxis (p = 0.99).

### Association of depressive symptoms with VTE recurrence in the multivariable analysis

In the aforementioned analyses, age, sex, SES, and unprovoked VTE were revealed as correlates of recurrent VTE with a significance level of p<0.20 and thus considered as covariates in the multivariable model. Table 2 shows the covariate-adjusted logistic regression model for the relation of depressive symptoms with the risk of VTE recurrence during the observation period. For a three-point increase in the level of depressive symptoms, there was a 44% increase in the relative risk of recurrent VTE, whereas sociodemographic factors and unprovoked VTE were not significantly predictive in their own right.

**Table 2:** Risk of recurrence of venous thromboembolism with depressive symptoms.

Entered variables	Odds ratio	95% CI	P-value
Age	1.019	0.986, 1.053	0.273
Male sex	1.088	0.433, 2.735	0.858
Educational level	1.740	0.907, 3.338	0.096
Unprovoked venous thromboembolism	2.478	0.899, 6.829	0.079
Depressive symptoms	1.444	1.015, 2.056	0.041

Depressive symptoms were entered in steps of 3 points. The model accounted for 10.5% of the variance (chi square = 13.99, df = 5, p = 0.016).

Because only two patients with recurrent VTE scored ≥8 on the HADS-D scale, there was limited power to test for a predictive value of this categorical measure of clinically relevant depressive symptoms for the risk of VTE recurrence. Therefore, we performed a median split on HADS-D scores yielding two groups of patients with relatively higher (5.52±2.73, range 3–16; n = 141) vs. relatively lower (1.01±0.75, range 0–2; n = 130) levels of depressive symptoms. After adjustment for age, sex, SES, and unprovoked VTE, the relative risk of developing recurrent VTE was 4.1-times higher in patients with higher compared to those with lower levels of depressive symptoms (OR 4.069, 95% CI 1.553, 10.664; p = 0.004).

In three complementary models, we adjusted for variables which associated with depressive symptoms in the correlation analysis with a significance level of p<0.20 in addition to age, sex, SES, and unprovoked VTE. After additional adjustment for obesity, OAC use during the observation period, and duration of the observation period, a 3-point increase in depressive symptoms remained a significant predictor of recurrent VTE, whereby increasing the relative risk by 44%, 57%, and 44%, respectively. The significance of results of the categorical analysis was also maintained with these adjustments (data not shown).

We also performed a sensitivity analysis with adjustment for age, sex, SES, and unprovoked VTE, for which we excluded the 6 patients who could not be objectively diagnosed with respect to recurrent VTE. For a three-point increase in the level of depressive symptoms, there was a 30% increase in the relative risk of recurrent VTE (OR 1.290, 95% CI 0.855, 1.946; p = 0.23). Moreover, the relative risk of developing recurrent VTE was 3.6-times higher in patients with more (range of HADS-D score: 3–16) than in those with less (range of HADS-D score 0–2) depressive symptoms (OR 3.629, 95% CI 1.267, 10.396; p = 0.016).

## Discussion

We found that depressive symptoms were significantly predictive for an increased risk of recurrent VTE in patients with a prior episode of VTE during a median observation period of about one year. This relationship was independent of demographic and clinical variables, which have also previously been shown to be associated with depression and/or the risk of recurrent VTE, and thus could have accounted for at least some of the increased risk of VTE recurrence with depressive mood. Expectedly, an episode of unprovoked index VTE was more frequent in patients with recurrent VTE than in those without VTE recurrence [12], while depressive symptoms showed the known association with obesity [25]. However, none of the covariates was revealed as an independent predictor of recurrent VTE above and beyond depressive symptoms in the multivariable model.

To our knowledge, our study is the first to show that a psychosocial factor is significantly associated with an increased risk of future recurrent VTE. Population-based studies have shown that psychosocial factors, including depressive symptoms, are prospectively related with incident VTE [4–7]. A parallel line of research on the etiology and prognosis of atherothrombotic diseases demonstrates that depressive symptoms are important for both incident and recurrent events [36, 37], even after controlling for sociodemographic variables, cardiac disease severity, and traditional cardiovascular risk factors [38]. The worldwide INTERHEART case-control study reported that perceived stress (due to work, home, finances or life events), and depression accounted for approximately one third of the population attributable risk of acute myocardial infarction [39]. Therefore, it is intriguing to assume that psychosocial variables, like depression, might as well account for a relevant portion of the risk of incident and recurrent VTE.

In our study, a 3-point higher level in depressive symptoms was associated with a 44% higher relative risk of VTE recurrence. In clinical terms, a patient with severe depressive mood, based on a HADS-D score of 14, would have an almost 1.5-fold higher risk of recurrent VTE than a patient with moderate depressive mood (HADS-D score of 11) and an almost 2-fold higher risk than a patient with mild depressive mood (HADS-D score of 8). We acknowledge that such implications are not beyond dispute, particularly regarding the risk inferred for more severely depressed patients. We found that only 10.0% of our patients scored in the range of clinically relevant depressive symptoms, whereby 7% had mild depressive mood and 3% had moderate or severe depressive mood. Nonetheless, the prevalence of a HADS-D score ≥8 in our study participants quite compares with the previously reported 15.5% of patients hospitalized with acute myocardial infarction meeting this cut-off [40]. And this even more so, as our patients were on average in a comparably less acute phase of the disease with studies showing, mental health is less impaired with longer time elapsed since the last VTE episode [41, 42]. Clearly, whether our findings are applicable to psychiatric patients and those with more severe depression remains to be seen. A retrospective study found a diagnosis of depression to be more frequent in psychiatric patients aged 65 or older who developed VTE during hospitalization than in those who did not, yet, only 5 out of 192 patients developed VTE in that study [43].

Because our study did not have the power to statistically test for an effect of clinically relevant depressive symptoms on the risk of VTE recurrence, we categorized HADS-D scores per cut-off in subclinical range of depressive mood. We found that levels of depressive symptoms not generally considered to be clinically relevant were associated with an increased risk of recurrent VTE. Specifically, independent of covariates, the group of patients scoring with at least 3 points on the HADS-D scale had an approximately 4-fold higher risk of future recurrent VTE relative to the group with a score of 2 or fewer points. The selection of this cut-off can be deemed arbitrary as it depends of the distribution of depressive symptoms in a given study sample. Nevertheless, the finding concurs with studies in patients with myocardial infarction showing that even minimal symptoms of depression significantly worsen the prognosis of the cardiac disease [35, 44].

We are not aware of any depression treatment studies in patients with VTE. However, the identification of potentially important psychosocial factors provides the possibility to intervene with mental health treatments to improve QoL [15] via alleviating depressive mood and perhaps even the risk of VTE recurrence. Counseling that includes advice from healthcare professionals on coping with problems (e.g., the fact of having experienced VTE) might be sufficient for the treatment of mild depression; moderately severe depression is generally responsive to psychotherapy, a physical exercise program, and/or antidepressants [45]. Regarding the latter, clinicians should be aware that SSRIs (e.g. paroxetine, fluoxetine) and SNRIs (e.g., venlafaxine, duloxetine) increase the risk of bleeding through interacting with OAC and platelet function [46]. In contrast, hypericum perforatum (St. John's wort) lowers plasma concentration of OAC drugs [47]. Moreover, tricyclics and antidepressants blocking serotonin receptors or with low potency of serotonin reuptake inhibition have been associated with an increased risk of VTE [48].

We mention several limitations of our study. We accounted for low SES that is associated with depression [24] and a wide range of poor healthcare outcomes [49], as well as for thromboprophylaxis and OAC during the observation period. However, depressive patients previously showed reduced OAC control [23] and compliance with medical treatment recommendations [50]. More objective measures, like percent time in the therapeutic range to monitor quality of OAC, should be considered in future studies. This would allow to test whether the impact of depressive symptoms on recurrent VTE is independent of poor OAC control or causally mediated through this mechanism. Inherent to observational studies, there remains a possibility of residual confounding through unrecognized and unassessed influencing factors. We cannot exclude a misclassification bias for our outcome variable. Depressive patients perceive their health as poorer than non-depressed ones [51]. Therefore, a recall bias regarding self-reports of VTE recurrence must be considered; this is of particular concern regarding the impossibility to objectify patient reports on VTE recurrence in 22.2% of cases. In fact, the relation between continuously (but not categorically) scaled depressive symptoms and the risk of VTE recurrence was attenuated to nonsignificance in a sensitivity analysis, for which these cases were excluded; however, this interpretation must consider reduced statistical power compared with the primary analysis.

Insufficient statistical power precluded stratified analyses in subgroups based on characteristics of the index VTE. For instance, the risk of future recurrent VTE may differ between subjects with one previous VTE event from those with several previous VTE events. Depressive patients tend to be more sedentary but we did not assess physical activity as a control variable that might account for some of the VTE risk. The results from this study might not generalize to psychiatric patients and geriatric populations with high comorbidity. We only studied patients who could be reached by phone, so we had no information available on the number of patients who died and the cause of death, including recurrent VTE. The exact duration of OAC therapy and number of patients on long-term OAC therapy could not reliably be verified from hospital charts, so we considered current use of OAC at study entry and anytime use of OAC during the observation period as potential covariates.

## Conclusions

We found that even subclinical levels of depressive symptoms after an index episode of VTE are significantly associated with the risk of recurrent VTE during a median observation period of about one year. In spite of its limitations, our study is of clinical interest and hypothesis-generating, as it is the first to suggest psychosocial factors may be important for VTE recurrence, so larger follow-up studies may be warranted. Also, the biobehavioral mechanisms linking depression, and perhaps other psychosocial factors with VTE recurrence, await further investigations.
